# Identifying evidence to define community-based rehabilitation practice in China using a case study approach with multiple embedded case study design

**DOI:** 10.1186/s12913-018-3838-7

**Published:** 2019-01-05

**Authors:** Eva Yin-han Chung

**Affiliations:** 1 Department of Special Education and Counselling, The Education University of Hong Kong, 10 Lo Ping Road, Tai Po, New Territories Hong Kong; 2 School of Occupational Therapy and Social Work, Curtin University of Technology, Perth, Australia

**Keywords:** Community-based rehabilitation, China, Program evaluation

## Abstract

**Background:**

This study examined community-based rehabilitation (CBR) practice in China on the basis of the development of an evaluation system using current evidence in a real context.

**Methods:**

A multiple embedded case study design was used to interpret both quantitative and qualitative data. In Part 1, a thematic analysis was conducted to identify the different levels of evidence available in CBR programs in China. Identified themes were then associated with the literature to form a system to code, categorise, and rank the obtained evidence. In Part 2, CBR practice was examined in 12 CBR programs by using the developed evaluation system with the CBR matrix and CBR framework.

**Results:**

Six themes under three categorised levels of evidence for demonstrating quality of practice were found. An evaluation system of program practice, based on existing standards to define levels of evidence, was created and used with the CBR matrix and CBR framework to evaluate the quality of practice in 12 CBR programs. The results of a within-case analysis revealed the strengths and areas for improvement in each program. An across-case analysis by using identified correlations revealed the characteristics of CBR practice in China, as demonstrated in the interaction of core CBR components. The content elements of CBR programs were significantly correlated with health outcomes, social development, education, and empowerment. Empowerment was significantly correlated with participant governance and community ownership.

**Conclusion:**

The proposed evaluation system, as developed in a real context, is feasible for monitoring and identifying the strengths of and areas for improvement in CBR programs. This study described the characteristics and interaction of different CBR components in CBR practice in China and is pertinent for enhancing the evidence-based practices and quality of CBR programs in China.

**Electronic supplementary material:**

The online version of this article (10.1186/s12913-018-3838-7) contains supplementary material, which is available to authorized users.

## Background

Community-based rehabilitation (CBR) is a multisectoral approach, aiming to equalise opportunities and include people with disabilities, while combating the vicious cycle of poverty and discrimination [1]. CBR is a common strategy used to promote the inclusion and participation of people with disabilities. It has evolved over nearly four decades, with practices varying in different countries and contexts [2]. However, the evidence base for CBR has not been well established in either Western or Eastern countries [3]. The effectiveness of CBR programs in China is rarely documented, and few studies have presented well-developed methods for evaluating CBR programs [4, 5], resulting in an incoherent and fragmented evidence base [6, 7]. Nevertheless, the systematic collection, analysis, and interpretation of information on the activities, outcomes, and quality of CBR programs are pertinent to constructing a strong scientific base for CBR and enhance its development.

The development of rehabilitation services in China has been evolving since its introduction in China in the early 1980s. In the early years, service providers in China were often volunteers with limited training in rehabilitation. With support from central and provincial organisations, grassroots rehabilitation communities have mobilised and coordinated local resources to meet the rehabilitation needs of people with disabilities [8, 9]. However, insufficient financial resources and personnel in rural communities, compounded by a lack of awareness from local authorities and the traditional hierarchical administrative system, have hampered the growth of CBR services in China [8]. Starting in 1991, the State Council approved three national 5-year work programs for people with disabilities, aiming to provide CBR services to all people with disabilities in Mainland China by 2015. Strengthening social support, capacity building in people with disabilities, the provision of a poverty alleviation fund, and the formation of local systems for the provision of services for people with disabilities are factors now expected to contribute to the success of CBR in the Chinese context [10]. Evidence of effectiveness can be found in the fact that poverty reduction among people with disabilities in China has declined, with the number of people with disabilities under the poverty line decreasing from 20 million in 1992 to 10.65 million by the end of 2004 [10].

CBR practice in China in the 1990s emphasized on medical treatment and the use of CBR stations as rehabilitation centres in districts [11]. The programs had been providing home-based training to people with disabilities. The first phase of CBR work involved a house-to-house screening survey, whereas the second phase involved the delivery of home-based functional training and the establishment of CBR stations equipped with modest therapeutic equipment. In the late 1990s, some nongovernmental organisations (NGOs) established CBR programs, which mainly work on medical and social rehabilitation. The success of these programs was identified as improved physical functioning, successful integration into schools, and vocational placement [12]. In the 2000s, the Ministry of Public Health established a partnership with a Hong Kong NGO and a key medical school in China to train doctors to implement CBR programs in their localities. This course was endorsed by the World Health Organization (WHO) and the China Disabled Persons Federation for training rehabilitation personnel as seeds for CBR development in China [13].

The evaluation of CBR programs in the Chinese context poses specific challenges. Although initial evaluative approaches were inspired from the Western methodologies, replicating Western models led to the exclusion of the cultural and linguistic uniqueness of Chinese communities, which influences the evaluation processes and results [14]. For example, using surveys in the Chinese CBR context is impractical because of the low literacy in rural villages, the large variety of dialects in Chinese communities, and difficulties in using Likert scales. Using Likert scales in Chinese culture is difficult because participants tend to select the midpoint [15] and skip some items [16]. This is because of the deeply held collectivist values within Chinese culture affect the self-reporting behaviour of Chinese people: Chinese people are expected to modestly rate their performance and refrain from exaggerating their achievements [17]. They are relatively conservative and encouraged not to openly discuss or comment on a person’s family or organisation [18]. The reluctance in China to evaluate service systems, programs or a family can result in the underrating of performance when using self-reporting scales [17]. However, when in-group pride, loyalty, and cohesiveness are focused on, a leniency bias in self-ratings is common for the sake of preserving the respect of the community [19]. These studies suggest that caution should be made regarding the reliability and validity of self-ratings and self-reports in the evaluation of Chinese CBR programs [20]. Experimental approaches can yield valid and reliable evidence, but these are considered unfeasible and unrealistic in the Chinese CBR context.

Appropriate veracity of evidence is required to evaluate programs, regardless of whether the comparison is to standards, other programs, or earlier periods. In a recent review of studies on CBR, only 4 of 114 identified studies described the evaluation of CBR programs in the Chinese context [21]. One study adopted a case study approach, which synthesised mostly documentary and self-reported data [22]. Two studies used interviews guided by questionnaires to collect self-reported data [23, 24]. The other was a randomised control trial, but its focus was on medical rehabilitation rather than CBR [25]. Thus, the use of self-reported and documentary evidence is most common in the evaluation of CBR; however, verified evidence is occasionally used.

Effective program evaluation is more likely to be achieved if a monitoring and evaluation system is adopted. CBR program evaluation assesses (1) the impact of a programs on people with disabilities and their families, (2) the impact of a program on a local community, (3) the quality of service, and (4) the quality of program management [26]. Complementary to evaluation, monitoring is a continuous function using the systematic data collection to provide management and main stakeholders an ongoing development intervention with indications of the extent of progress and achievement of objectives as well as the progress in the use of the allocated funds [26]. Numerous frameworks have provided well-defined levels of evidence [27–29]. Then following three main audit evidence types: oral, a verbal description or subsequent oral representation of events; documentary, a contemporaneously written record; and verified, a statement prepared by a credible third party (or an expert) to support claims. In these audit standards, oral evidence is considered less reliable, whereas documentary and verified evidence is considered more reliable. The oral presentations made by the assessed party would be regarded as relatively unreliable because of their oral and internal nature. Documentary or verified evidence should be included to support such oral claims or conclusions to ensure consistency and validity [28]. Documentary evidence refers to both internal and external documentation. Verified evidence denotes written or oral confirmations from an independent third party, and such confirmations can be positive or negative [30].

The WHO has developed a conceptual framework for CBR called the CBR matrix [31]. It reflects a comprehensive multisectoral approach to CBR. The CBR matrix consists of five components: health, education, livelihood, empowerment, and social. The WHO has also developed the Community-based Rehabilitation Indicators Manual to measure the impacts of a program on a community in terms of achieving the outcome indicators [1]. However, the indicator manual cannot measure the qualitative impact of a CBR program on the lives of people with disabilities, local community, service quality, or program management.

The CBR Framework [22] is a framework with potential to understand and improve the quality of CBR practice in Chinese communities. The framework comprises 5 domains, 25 categorised core elements, and 72 indicators. The five domains are participant outcomes, CBR program content, participant empowerment, community ownership and governance, and program operation and management. It provides a full set of indicators for each element to capture the quality of practice [22].

This study identified and used appropriate types of evidence to evaluate and describe the quality of CBR practice in China. The objectives of this study were as follows:

1. Identify and quantify the types of evidence presented in Chinese CBR programs to indicate best practice (part 1).
2. Evaluate and describe CBR practices in China using the identified types and levels of evidence, with the CBR matrix and CBR framework (part 2).

## Method

### Part 1: Identifying and ranking types of evidence commonly available in Chinese CBR programs

To meet the first objective, this study used a case study methodology to explore and define the common types of evidence in CBR programs in China. The case study approach is used in qualitative research to explore a case or a system through in-depth data collection involving multiple sources of information [32]. The case study methodology used here was described by Eisenhardt [33], comprising five steps, namely commencing, selecting cases, crafting instruments and protocols, field study, and analysing data. The research question is defined in the first step, presented above.

### Selecting cases

Five CBR programs in China were selected. To be selected, the CBR programs needed to meet three of the following five criteria: (1) focusing on the provision of rehabilitation activities for people with disabilities [4, 31, 34]; (2) working with a community to ensure inclusion of people with disabilities [35]; (3) addressing poverty experienced by people with disabilities and their families [4, 31]; (4) working on self-help and mutual-help issues among people with disabilities or organisations for people with disabilities [36]; and (5) advocating and concentrating on equal participation of people with disabilities within society [31]. CBR programs located in urban or rural areas with different funding sources (government departments, NGOs, and international organisations) were identified through the professional network of the investigator and leaders of known CBR programs and by conducting a search for appropriate programs on the Internet. This study was granted ethical approval by the Human Research Ethics Committee at Curtin University (Reference Number HR 68/2007).

### Crafting instruments

To guide data collection and ensure consistency, a case study protocol was developed, which included an overview of the study, field procedures, case study questions, and a guide for reporting the case study.

### Entering the field and data collection

The objective of this stage was to identify the type and content of evidence routinely available in CBR programs in China to determine the quality of these programs. Data collection methods used in this phase included interviews, observations, and reading relevant documents (Table 1). A detailed examination of each program was conducted during a 4-day field visit. Semistructured interviews were conducted with program managers, CBR workers, and service users (in the form of home visits or activity programs). Case study questions were designed to guide the interviews (Additional file 1). The following information was collected for each program: (1) objectives, expected outcomes, rationale, and operational processes; (2) activities conducted; (3) evidence of achieving the stated outcomes; and (4) evaluative procedures.

**Table 1:** Data collection methods and procedures in each program

Method	Procedures
Interviews	Interview with Program Manager(s) Interview with 2–4 CBR workers Interview with 6–8 service users Interview with other concerned parties, if any.
Reading documents	Examine program-related documents Examine participant-related documents Examine external reports and documents, if any.
Observations	Observe in 6–8 home / community visits Observe in activity programs, if any.
	Observe in meetings, if any.

### Thematic analysis

All interviews were transcribed. Observations and findings from the document review were recorded in a field visit report. The analysis of the transcripts and documents was completed in the original language (Chinese). Using the full set of transcripts and field visit reports, types of evidence to support best practice were identified. A thematic analysis was completed in the following six steps [37]: (1) familiarisation with the data, (2) generation of initial codes, (3) identification, (4) evaluation, (5) definition of themes, (6) and writing of the report. After the collected raw data in the five programs were read, data regarding program evaluation and descriptions of best practice were coded. These codes represented the most basic segments of information that could be assessed meaningfully with reference to evaluating CBR practice [38]. The relationships between the themes and codes regarding program evaluation were examined, and a thematic map of analysis was generated. Clear definitions of the themes were subsequently generated from the obtained evidence.

### Part 2: Evaluating and defining CBR practice in China using legible evidence collected from the field

To identify CBR practice in China, the research team used the newly identified system to collect evidence from 12 CBR programs. The case study approach was adopted in Part 2 as well. This study proposed that the quality of CBR practice in China could be documented and defined by using the newly developed ranking system on levels of evidence. Evaluation of service quality is defined as identifying evidence to reveal a program’s functional quality, process of service delivery, service outcomes and the reliability, responsiveness, empathy, assurance and tangibles associated with a service experience [40]. Quality of practice is not defined as “zero defects” or “conformance to requirements” here in the Chinese CBR context. Rather, quality of CBR programs was examined in this study for getting insights for improvement actions in case of quality shortfalls [39].

This stage employed an embedded case study design because it allowed the investigator to study the programs through preselected categories of descriptions as the focus for the study [41]. The preselected categories of the descriptions were three levels of evidence along with the components in the CBR framework and the CBR matrix. The CBR matrix is a framework comprising 5 domains and 20 elements [31]. The CBR framework consists of 5 domains and 25 elements. A full set of indicators were developed in both the CBR matrix [1] and CBR framework [42], which were used in this study to define the CBR components for pattern matching.

### Case selection

Seven CBR programs were added to the original five programs in Part 1. In total, 12 CBR programs were recruited following the inclusion criteria detailed in Part 1. Data gathered from the original five programs, along with those collected from the new programs, were subsequently analysed.

### Crafting instrument

#### Case study protocol

The protocol design and data collection procedures were identical to those in Part 1. Data collection methods were the same as for the first study.

### Entering the field and data collection

A 4-day field visit was again conducted for each program, as in Part 1.

### Data analysis

All interviews were tape-recorded, and all observations in the field visits were recorded in the field-visit report. Data were recorded as a Microsoft Access file. Evidence of CBR components, as reflected by the respective indicators, in each program was coded and charted according to the elements and domains in the two frameworks. The number of coded themes categorised as oral evidence, documentary evidence or verified evidence was entered into a program summary table. This process was overseen by an adviser and the research supervisor.

### Within-case analysis

The collected data were linked to the propositions by formulating two program profiles for each program, one using the CBR matrix and the other using the CBR framework, in the form of a case description matrix for pattern matching. By developing a case description matrix for the programs, evidence was placed within different categories. Pattern-matching techniques were used for relating data to propositions [43]. Through simple pattern matching, the information found was matched to each category (i.e., each element), as suggested by the two frameworks. Data were matched against the indicators to detect elements supported by the corresponding types of evidence. The coded themes, as supported by documentary or verified evidence, were counted and entered into an indicator summary table. Based on the coding of elements under the two frameworks, the profiles of the 12 programs examined using the two frameworks were revealed.

### Across-case analysis

With the quantified evidence (shown in the program summary table) obtained from the two frameworks, the characteristics of CBR practice in China, as revealed by the framework, were compared to define their nature and quality. The number of coded themes under each category was converted to a percentage score to enable comparison of patterns across programs. The practices of Chinese CBR programs were revealed through the integration of results from all programs and using correlations to identify the relationships among the components as reflected through the CBR matrix and CBR framework.

## Results

### Part 1: Identifying and ranking types of evidence

Overall, 5 top managers, 5 program managers, 4 deputy managers, 42 people with disabilities, 23 family members, 18 CBR workers, and 7 county officials participated in this study. The research team conducted 35 home visits and observed 8 activity programs in five CBR programs.

The six identified types of commonly cited evidence were (1) description of processes, (2) description of outcomes, (3) description of examples, (4) presenting examples, (5) citing program-related documents, and (6) citing case-related documents. The frequency of occurrence of evidence describing practice within each theme was examined by counting coded data in the data material collected from all five programs (Table 2). The most common type of evidence found in the field was oral descriptions of the CBR process (37.36%). In general, people running the programs communicated to others how their CBR processes could effectively meet the needs of people with disabilities; they also showed real examples (23.69%). To demonstrate best practice, program managers selected clients and their family members to meet the investigators to detail the changes in the person or community through the CBR processes. The least common type of evidence used was case-related documents (1.14%). Case records were present in some programs, but the content mainly comprised personal details and medical histories of the service users. Objective documentation of the progress of treatment or activities was rarely seen. In summary, the most common type of evidence was description of process, whereas case-related documents was the least common. Showing real examples was more common than citing documents to support best practice.

**Table 2:** Types of Evidence Supporting Best Practice

	Program					
	1	2	3	4	5	Total (%)
Description of process	49	27	18	41	29	164 (37.36)
Description of outcomes	9	7	7	21	13	57 (12.98)
Description of examples	7	8	3	4	2	24 (5.47)
Showing real examples	27	17	16	23	21	104 (23.69)
Citing program-related document	17	11	11	25	21	85 (19.36)
Citing case-related document	0	0	1	4	0	5 (1.14)

Note. number of counts is the counting of coded themes as support by documentary or verified evidence

### Ranking levels of evidence

Once the types of evidence were identified, related studies provided a rationale for organising and ranking the order of the different types of evidence. Following the literature, four principles guided the ranking process:

1. Documentary evidence is more reliable than oral evidence [28]
2. Evidence seen by a third party is more reliable than quoted by a party within an organisation [29]
3. Evidence generated through the investigator’s direct observation is superior to trusting oral descriptions [29]
4. Verified evidence produced by an expert is reliable [28].

The six types of commonly found evidence were organised according to this ranking to form three levels of evidence: (1) oral evidence (descriptions of processes, outcomes, and examples), (2) documentary evidence, and (3) verified evidence. Citing programs or case-related documents was considered a form of documentary evidence, whereas showing real examples (provided the investigator is considered a credible third party) met the definition of verified evidence. On the basis of these principles, oral evidence was regarded as insufficient support for best practice; documentary and verified evidence was regarded as suitable support for best practice.

### Part 2: Evaluating and defining of CBR practice in China

Seven additional programs were recruited in the second study, along five from the first; together, 12 programs were included. These programs varied in terms of background, funding sources, location, and nature. Among these programs, four were in rural areas and the other eight were in urban areas. Three programs were funded by local government, two by international humanitarian organisations, and three by organisations for people with disabilities; the remaining four were operated by local nonprofit (philanthropic) organisations.

### Within-case analysis (Table 3)

Program 1 was a rural program funded by local government. It provided physical and social rehabilitation services, emphasised improving participant outcomes, and possessed a reasonable number of CBR-related program content elements. The program mainly focused on improving health outcomes and advocating empowerment.

**Table 3:** Program Summary Table

	Program											
	1	2	3	4	5	6	7	8	9	10	11	12
CBR Framework												
Participant Outcome	16	11	14	13	13	7	7	22	9	9	11	8
CBR Program Content	16	11	10	19	18	5	6	20	2	4	10	3
Participant Governance	2	0	0	5	7	3	1	4	4	2	4	2
Community Ownership	4	1	1	6	2	2	1	7	3	1	1	2
Program Operation & Development	7	6	6	8	3	11	5	16	9	6	7	5
CBR Matrix												
Health	11	4	8	5	10	3	4	13	8	7	6	7
Education	2	2	0	5	0	0	1	2	0	0	2	0
Livelihood	3	2	1	2	0	3	1	3	0	0	0	0
Social	6	7	8	11	7	3	2	10	2	4	10	4
Empowerment	13	4	6	19	18	8	10	22	8	3	8	2

Note. number of counts is the counting of coded themes as support by documentary or verified evidence

Program 2 was a foster care program funded by local government. It aimed to improve the health status of abandoned children with disabilities and placed them with families. It was strongest in improving participant outcomes and the social status of the children with disabilities. Participant governance was not a major focus, and the program adopted a top-down approach to decision making.

Programs 3 and 4 were operated by an international humanitarian organisation. They were located in different provinces. Program 3 was based on a rehabilitation centre, whereas Program 4 was based on community outreaching teams. Both programs focused on raising awareness. Program 3 was the strongest in improving participant outcomes, with its focus being on health and social components. Program 4 was well-developed in the sense that it also achieved social and empowerment components. It was evident that this program possessed a good amount of CBR-related content elements.

Programs 5, 7, and 11 were organisations for people with disabilities, all of which emphasised the improvement of psychosocial outcomes of people with disabilities and their families. Program 5 worked with adults with physical disabilities, mainly focusing on improving the components of health and empowerment, but did not enhance the education and livelihood of people with disabilities. The emphasis in Program 7 was on advocating autonomy and independence for people with physical disabilities. Program 11 was a mutual-help group for children with disabilities and their parents. This program focused on improving the social components and the functional outcomes of participants.

Program 6 was a community-based vocational rehabilitation program, offering sheltered employment to adults with physical and mental disabilities. It worked on the component of empowerment through the development of job skills and the potential of participants. The results revealed that the operation and management of this program were effective.

Program 8 was a home-based program in an urban district operated by an NGO. It performed well at improving the functional outcomes of adults with disabilities and their families and focused on health, social, and empowerment, as presented in the CBR matrix.

Program 9 was a community-based program promoting independent living. It focused on improving health and empowerment, but not on the education and livelihood of people with disabilities.

Finally, Programs 10 and 12 were rehabilitation centres. They worked to improve the functional outcomes and health status of people with disabilities.

### Across-case analysis

The percentage profile (Table 4) showed the comparison results among the 12 programs. Programs 8 and 4 were the programs that demonstrated the most CBR elements. Integration of results from all programs revealed that the most commonly reported best practice was in the domain of participant outcomes and empowerment. The least reported domains were education and livelihood. Correlation results are shown in Table 5.

**Table 4:** Percentage profile of coded themes on CBR elements of the twelve programs

	CBR Framework					CBR Matrix					
	Participant Outcome	CBR Program Content	Participant Governance	Community Ownership	Program Operation & Development	Health	Education	Livelihood	Social	Empowerment	Total
P1	2.20	2.20	0.27	0.55	0.96	1.51	0.27	0.41	0.82	1.79	10.99
P2	1.51	1.51	0.00	0.14	0.82	0.55	0.27	0.27	0.96	0.55	6.59
P3	1.92	1.37	0.00	0.14	0.82	1.10	0.00	0.14	1.10	0.82	7.42
P4	1.79	2.61	0.69	0.82	1.10	0.69	0.69	0.27	1.51	2.61	12.77
P5	1.79	2.47	0.96	0.27	0.41	1.37	0.00	0.00	0.96	2.47	10.71
P6	0.96	0.69	0.41	0.27	1.51	0.41	0.00	0.41	0.41	1.10	6.18
P7	0.96	0.82	0.14	0.14	0.69	0.55	0.14	0.14	0.27	1.37	5.22
P8	3.02	2.75	0.55	0.96	2.20	1.79	0.27	0.41	1.37	3.02	16.35
P9	1.24	0.27	0.55	0.41	1.24	1.10	0.00	0.00	0.27	1.10	6.18
P10	1.24	0.55	0.27	0.14	0.82	0.96	0.00	0.00	0.55	0.41	4.95
P11	1.51	1.37	0.55	0.14	0.96	0.82	0.27	0.00	1.37	1.10	8.10
P12	1.10	0.41	0.27	0.27	0.69	0.96	0.00	0.00	0.55	0.27	4.53
Total	19.23	17.03	4.67	4.26	12.23	11.81	1.92	2.06	10.16	16.62	100.00

**Table 5:** Correlations of elements in the twelve programs

	Participant Outcome	Program content	Participant governance	Community ownership	Program development	Health	Education	Livelihood	Social	Empowerment
Participant Outcome	1.000									
Program content	.785	1.000								
Participant governance	.199	.315	1.000							
Community ownership	.434	.410	.601	1.000						
Program development	.221	.150	.316	.572	1.000					
Health	.729	.319	.268	.477	.064	1.000				
Education	.456	.687	.132	.302	.333	−.063	1.000			
Livelihood	.338	.527	−.153	.437	.550	−.050	.495	1.000		
Social	.745	.778	.306	.210	.187	.239	.616	.199	1.000	
Empowerment	.518	.748	.619	.662	.370	.340	.518	.458	.383	1.000

## Discussion

### Use of real evidence to form an audit standard

This study identified the use of commonly available evidence in the Chinese CBR context to describe CBR practice in China. The identified evidence was related to audit standards to form a program audit system to understand CBR practice. Along with the CBR matrix and CBR framework, it can be used to monitor and enhance the quality of CBR programs. One advantage of using such an audit system is that it encourages program operators to think of actual evidence to achieve program outcomes. Only having a process in mind or telling a story of change is insufficient for demonstrating best practice. Best practice must be supported with real-world examples, proper written records, or scientific evaluation reports.

The terminology used in the proposed audit system is easy to understand. Evidence to support best practice is categorised under three levels with different subthemes: (1) oral, description of processes, outcomes, and examples; (2) documentary, citing case and program documents; and (3) verified, showing real examples and scientific evidence. The terminology was derived from analysing real practice and has resulted in user-friendly terms that can be understood even by workers with a lower level of education. This is relevant and appropriate in the CBR field because community members are mobilised and trained to work with people with disabilities. It encourages the realisation that a proper records or real examples are vital for supporting program achievement.

### Nature of CBR practice in China, as reflected in the within-case analysis

CBR practice in China was revealed in the within-case analysis. Together with the CBR matrix, the analysis showed that most programs promote empowerment and health among people with disabilities. When analysed with the CBR framework, it was evident that most programs (11 of 12) provided sufficient evidence of enhancing participant outcomes. Two-thirds of the programs focused on enhancing the CBR content elements advocacy, networking, family member involvement, neighbours or community member involvement, and CBR personnel development. The obtained documentary and verified evidence confirmed that the programs were developing the CBR program elements. Both the CBR matrix and CBR framework revealed that these Chinese CBR programs developed beyond the provision of health and rehabilitation services to include more CBR elements to enhance the inclusion and participation of people with disabilities.

In the 1980s, CBR programs in China were mostly initiated by medical doctors or rehabilitation therapists and operated within the health or social service sectors of local government. They emphasised the outcomes obtained from the provision of appropriate interventions, originally based on a medical model [9]. Enhancing health outcomes remains a major objective in CBR practice in China. The work of rehabilitation in Chinese CBR programs combined modern rehabilitation techniques with traditional Chinese techniques; rehabilitation as rendered by rehabilitation personnel were provided in rehabilitation institutions whereas community-based support programs were relying on families of people with disabilities [13]. Different from traditional medical rehabilitation programs, CBR programs aim to enhance networking, advocacy, and participant empowerment. With the development of CBR in China during the next two decades, most programs realised these goals and worked towards promoting empowerment in people with disabilities through the mobilisation of local community resources [3]. With the efforts of the central government through the China Disabled Persons Federation, various levels (district, county, and street) work to organise community service networks, medical prevention and health care networks, organisations for people with disabilities, and other social forces to conduct CBR work [13].

However, the results of this study show that the components of education and livelihood are not the focus in CBR practice in China. Educational service is operated under the Ministry of Education of the People’s Republic of China. CBR programs in China work to facilitate the integration of children with disabilities into the education system. However, this is frequently limited by architectural barriers, the remoteness of communities, and the difficulties in handling children with severe disabilities. Projects enhancing livelihood in CBR programs were evident [44], but they were isolated activities that have not been fully developed and extended to other areas for people with different types of disabilities [13].

The results of this study show that community participation was not commonly seen in programs. Community participation includes the involvement of people with disabilities in CBR programs, from policy making to implementation and evaluation. Participant governance and ownership in CBR programs were directed by the central government rather than by people with disabilities. Financial support from the central government is regarded as a vital sign of community ownership of programs in China. The program organisers had to set aside their individual agenda and put forward objectives stated by local government.

### Interaction of components as shown in the across-case analysis

Comparison of programs and the interaction of CBR components was revealed in the across-case analysis. The commonly reported element among the 12 programs was participant outcomes. The content elements in CBR programs were found to be significantly correlated with participant outcomes, health enhancement, social development, improved education, and empowerment. This finding demonstrates the importance of a comprehensive CBR program design to achieve designated CBR outcomes. Such a comprehensive design follows the elements in the domains of the CBR framework [22]: advocacy, networking, involvement of family members of people with disabilities, involvement of neighbours and community members, development of CBR management, and CBR workers.

Significant correlations were also found between the components of empowerment and community ownership and between empowerment and participant governance. Again, this highlights the importance of advocacy, community mobilisation, promoting self-help and mutual-help in the process of empowerment. Community ownership is the actualisation of the rights of people with disabilities. People with disabilities have the right and autonomy to take leadership roles and participate in the community. The process of empowerment clearly interacts with the participant governance and community ownership of programs. Overall, 8 of 12 program organisers provided evidence that they promoted empowerment; however, only a few of them could provide sufficient evidence of attaining community ownership and participant governance. Participant governance denotes a change in traditional power structures within programs or the community [4]. The Chinese interpretation of human rights in relation to political power is different from the Western perspective. Traditional Chinese culture values stability and harmony. According to the rule of propriety, respecting authorities in accordance with the structures of various social systems brings harmony [45]. Therefore, radical actions that advocate societal change are less common in Chinese communities. Irrespective of what is right or wrong, different stakeholders within CBR systems conform to the government and act in accordance with the expectations of the local communities to ensure stability. This behaviour might be criticised by Western countries as fostering compliance to local government. Democracy in China is a controversial issue. The contours of Asian democracy are completely different from those of contemporary American democracy; therefore, serious problems may occur when reconciling individual rights with the interest of a collective community [46].

## Limitations

The use of a case study approach to build evidence for program evaluation has limitations. The extensive use of empirical evidence can produce a high level of complexity and confusion. This study extensively examined the practices of 12 programs. A considerable amount of information regarding CBR practices in context was collected. This type of approach required a clear and strong framework to prevent a loss of focus while processing the data. Furthermore, a scientific approach to CBR evaluation may not be appealing sometimes because people may think the voices of people with disabilities, which could not be supported with documentary or verified evidence, could not be well-acknowledged in the process of evaluation.

## Conclusion

A qualitative methodology was used to identify the possible types of evidence available and the feasibility of supporting the practices in CBR programs in China. Themes regarding levels of evidence for best practice were compared with audit standards to delineate a measurement system to describe practice. Used with the CBR matrix and CBR framework, the measurement system provides a mechanism for identifying the strengths and areas for improvement in various programs. It also enables evidence-based practice because formal documentation and the provision of verified information are encouraged when evaluating a program. Evidence-based practice for CBR programs in Chinese communities were explored and documented to enhance further development of CBR in China.

## Additional file

Additional file 1: Case study quesions (DOCX 18 kb)

## Abbreviations

CBR: community-based rehabilitation
NGO: non-governmental organizations.
WHO: World Health Organization
