# Supplementary material for: Identifying evidence to define community-based rehabilitation practice in China using a case study approach with multiple embedded case study design
Source: BMC Health Serv Res. 2019 Jan 5;19:6. doi: 10.1186/s12913-018-3838-7 (PMC6321712; doi:10.1186/s12913-018-3838-7)
Supplement: Supplementary file 1 — Case study quesions (DOCX 18 kb) [file 12913_2018_3838_MOESM1_ESM.docx]

Additional file 1. Case study questions

*Questions for the program managers*

1. Introduction of the program.
2. What is the clientele of your program?
3. How can a PWD know and join your program?
4. Description of the procedures of entering and exit of your program by the service user?
5. What will your program do to a service user after entering your program?
6. What are the objectives of the program?
7. How the objectives can be achieved in the program?
8. What are the common activities of the program?
9. Where do the activities commonly take place?
10. How do the activities operate?
11. What are the expected outcomes from the activities?
12. Is there any linkage / cooperation with other counterparts in the community?
13. How can your program be supported by the community / government?
14. How can your program assert the rights of the PWD in the community? Any activities on advocacy carried out in the past year?
15. What are the successful outcomes of your program?
16. Any service standards set and accomplished by the service?
17. How do deal with the ethical issues during service delivery?
18. Power distribution among service user and staff.
19. Account for the reasons for the success.
20. What are the roles and functions of different staff and volunteers in the program?
21. Any directions for future development?
22. Any research activities or staff training and development activities?

*Questions for the program users (the PWD)*

1. Background of the program users.
2. History of engaging in the program.
3. Perceptions towards the program – its objectives, functions and roles in the community
4. Service received / participation in the program activities
5. Any change in functional level brought by the program? How?
6. Any change in social participation brought by the program? How?
7. Any change in education or financial status brought by the program? How?
8. Sense of belonging and commitment to the program
9. Perceived sense of autonomy in the program
10. Perception on the staff of the program
11. How can the program help the users in fighting for their rights in the community?
12. Overall satisfaction level with the program.
13. Any appreciation or suggestions for the program.
14. What is your relationship with the neighbours and other community members?
15. Do you have sufficient support from the community and government to sustain your living in the community?

Points to Note in reading of Documentation (program leaflet, newsletters, annual report, case notes and other publications)

1. Objectives of the program
2. Common activities in the program
3. Any change in functional status of the program user (the PWD), in which dimensions, how?
4. The relationship of the program with the community – any donations, funding source
5. Expected outcomes from the program.
6. Any explicit indicators for the outcomes.
